# Correction: Novel pyrimidine linked acyl thiourea derivatives as potent α-amylase and proteinase K inhibitors: design, synthesis, molecular docking and ADME studies

**DOI:** 10.1039/d5ra90008e

**Published:** 2025-01-23

**Authors:** Hina Zaman, Aamer Saeed, Hammad Ismail, Sadaf Anwaar, Muhammad Latif, Muhammad Zaffar Hashmi, Hesham R. El-Seedi

**Affiliations:** a Department of Chemistry, Quaid-I-Azam University Islamabad 45320 Pakistan asaeed@qau.edu.pk +92-51-9064-224 +92-51-9064-2128; b Department of Biochemistry and Biotechnology, University of Gujrat Gujrat 50700 Pakistan hammad.ismail@uog.edu.pk; c Department of Biological Sciences, International Islamic University Islamabad 45500 Pakistan sadaf.anwaar@iiu.edu.pk; d Centre for Genetics and Inherited Diseases (CGID), Taibah University Al-Madinah Al-Munawwarah Kingdom of Saudi Arabia latifmayo@yahoo.com; e Institute of Molecular Biology and Biotechnology, The University of Lahore Pakistan muhammadzaffar.hashmi@fulbrightmail.org; f Department of Chemistry, Faculty of Science, Islamic University of Madinah Madinah 42351 Saudi Arabia elseedi_99@yahoo.com

## Abstract

Correction for ‘Novel pyrimidine linked acyl thiourea derivatives as potent α-amylase and proteinase K inhibitors: design, synthesis, molecular docking and ADME studies’ by Hina Zaman *et al.*, *RSC Adv.*, 2024, **14**, 33235–33246, https://doi.org/10.1039/D4RA05799F.

The authors regret that one of the affiliations (affiliation *e*) was incorrectly shown in the original manuscript. The corrected list of affiliations is as shown here.

The Royal Society of Chemistry apologises for these errors and any consequent inconvenience to authors and readers.

